# Supplementary figures and images for: m6A RNA methylation facilitates pre-mRNA 3’-end formation and is essential for viability of Toxoplasma gondii
Source: PLoS Pathog. 2021 Jul 29;17(7):e1009335. doi: 10.1371/journal.ppat.1009335 (PMC8354455; doi:10.1371/journal.ppat.1009335)

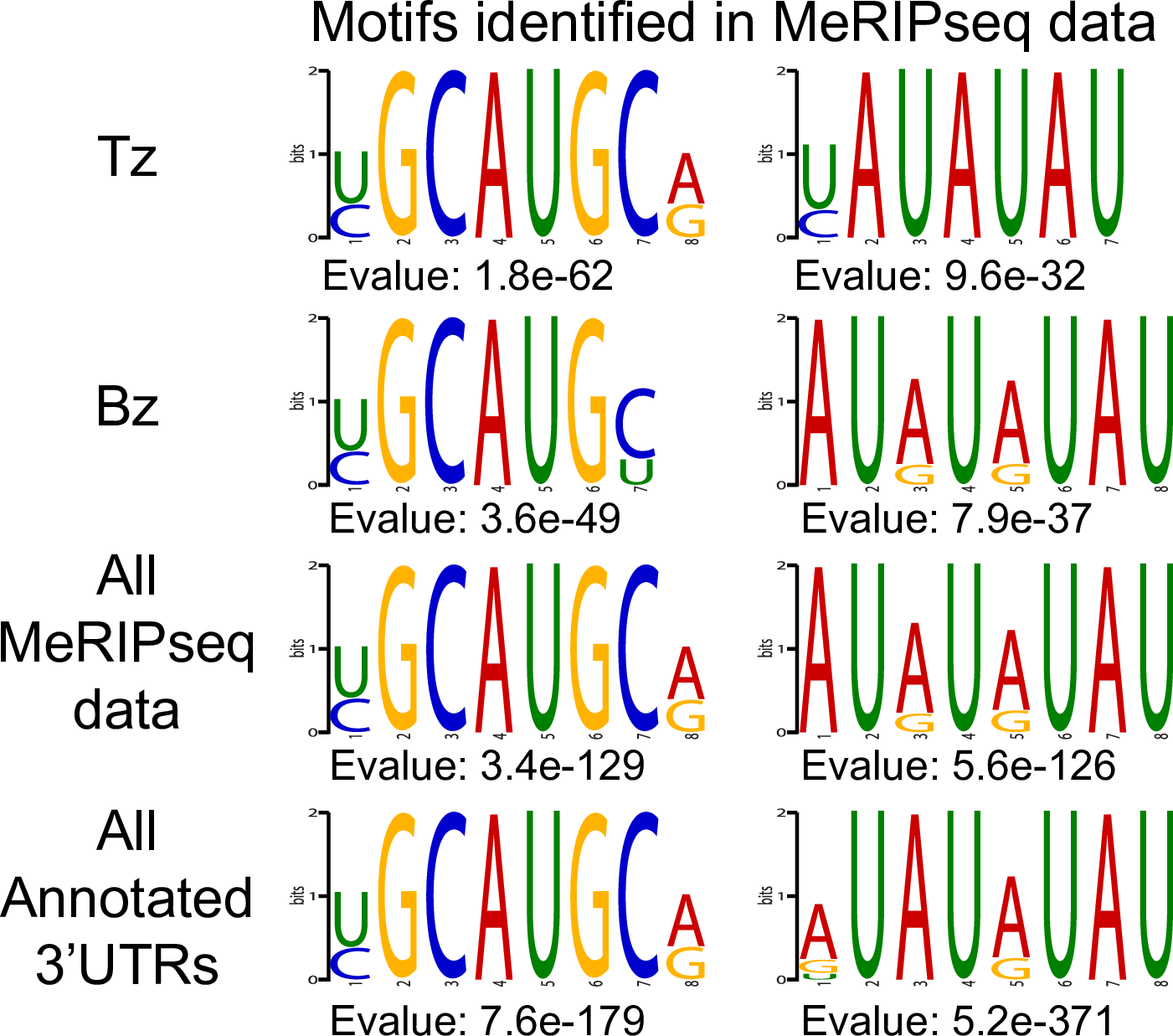

Supplement: S1 Fig — Significance (e-value) is also displayed. (TIF) [file ppat.1009335.s001.tif]

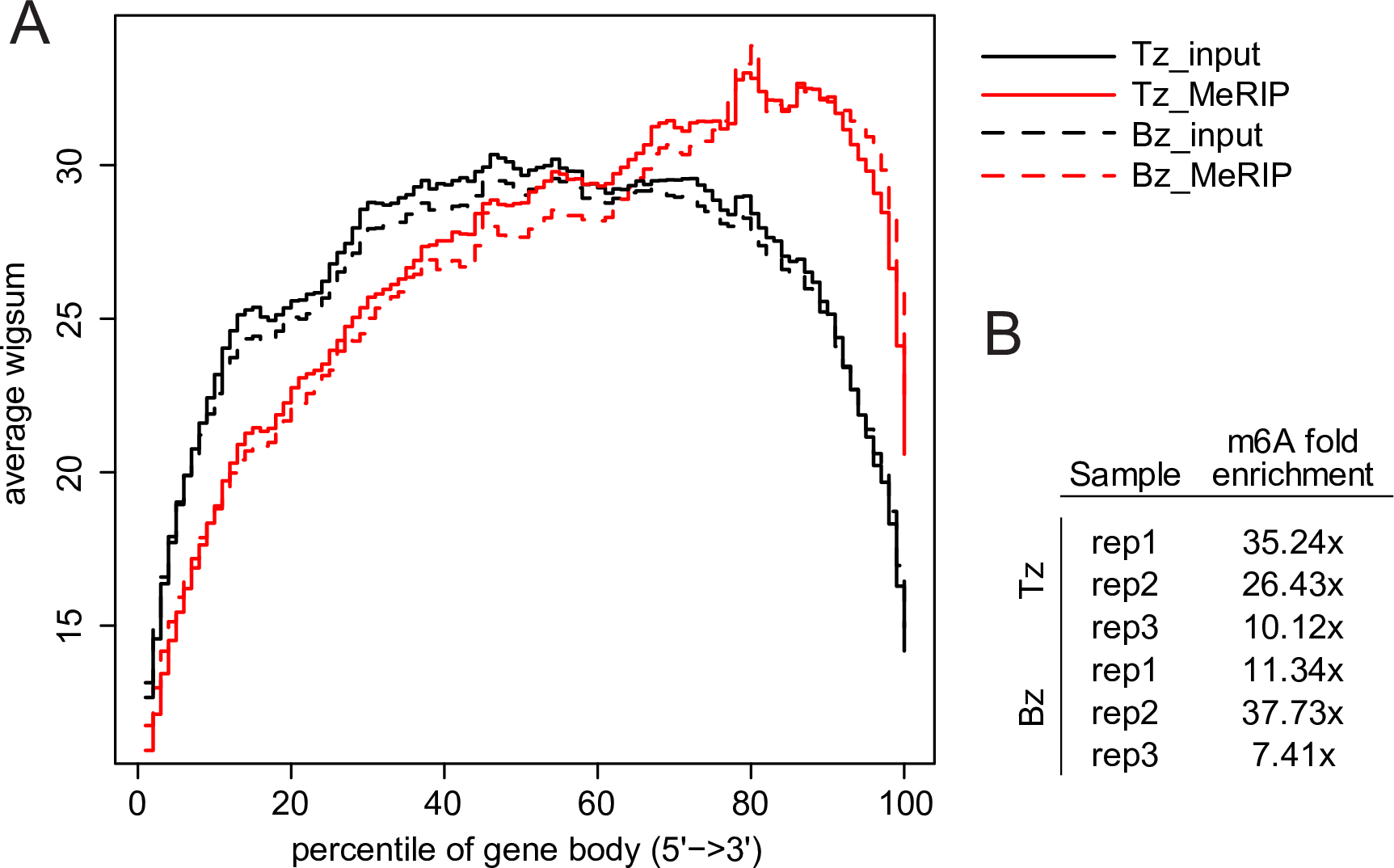

Supplement: S2 Fig — A) Metagene plot showing coverage of input (black) and m6A-enriched samples (red) from tachyzoite (solid lines) and bradyzoite-induced (dotted lines). B) Estimate of fold enrichment after m6A immunoprecipitation. (TIF) [file ppat.1009335.s002.tif]

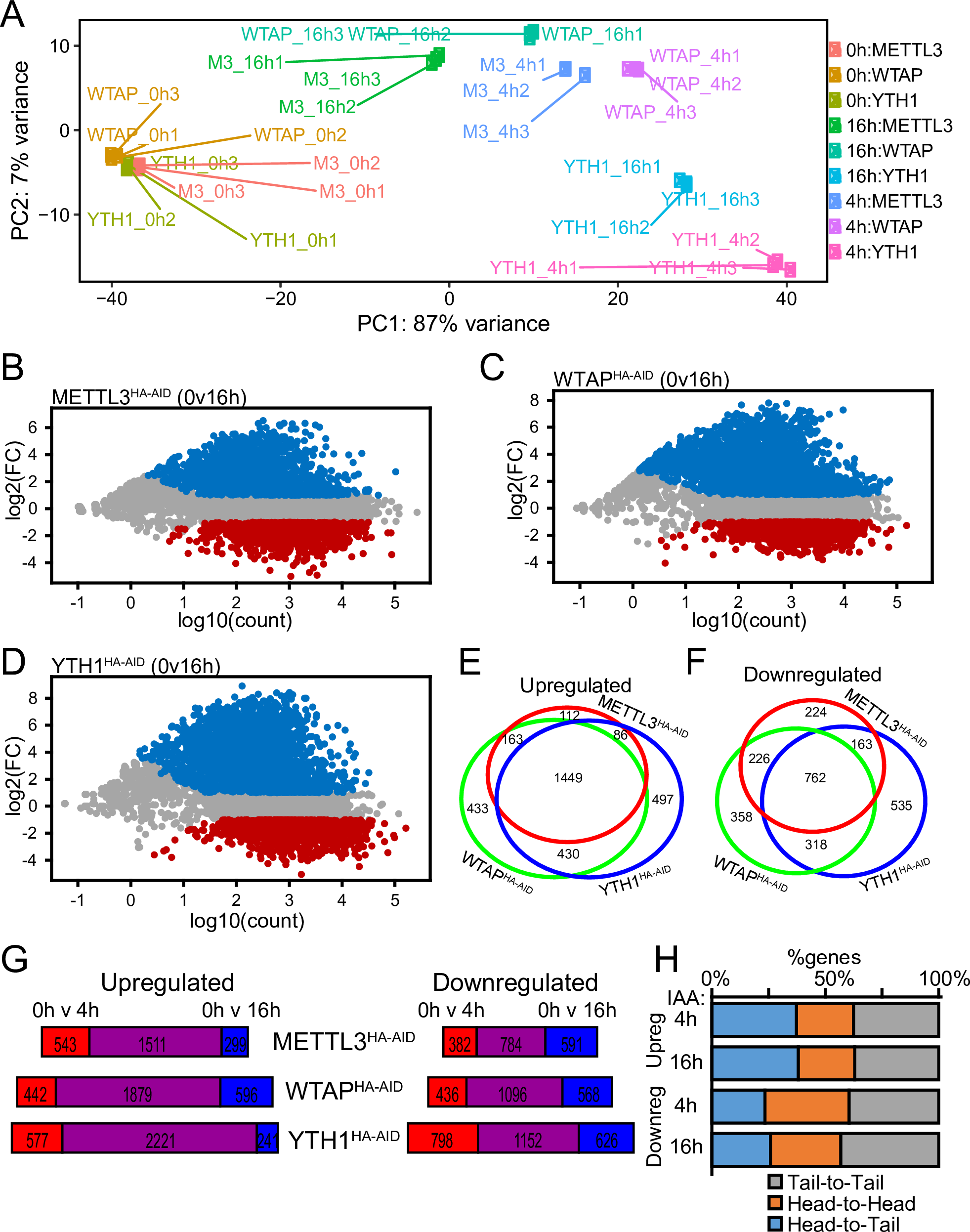

Supplement: S3 Fig — A) Principle component plot showing variance between replicates and all samples. B-D) Differential gene expression analysis after 16 h treatment with 500 μM IAA for METTL3HA-AID (B), WTAPHA-AID (C) and YTH1HA-AID (D) parasites. E-F) Venn diagram showing the relationship of upregulated (E) and downregulated (F) differentially expression genes between lines after 16 h IAA treatment. G) Diagrams showing relationship between differentially expressed genes after different IAA treatment times within strains. H) Analysis of gene orientation for shared upregulated and downregulated genes after IAA treatment. (TIF) [file ppat.1009335.s003.tif]
